# Association Between a Body Shape Index and Epilepsy Among US Adults: Potential Indirect Pathway Through Depressive Symptoms

**DOI:** 10.1002/brb3.71625

**Published:** 2026-07-28

**Authors:** Qiaoduan Feng, Xiujuan Mi, Hongbin Tang, Shaokun Yang, Can Wan, Jinsong You, Jun Tang

**Affiliations:** ^1^ Department of Encephalopathy Chongqing Traditional Chinese Medicine Hospital Chongqing China; ^2^ The Second Clinical College of Guangzhou University of Chinese Medicine Guangzhou China; ^3^ Department of Epidemiology and Biostatistics, West China School of Public Health and West China Fourth Hospital Sichuan University Chengdu China; ^4^ Department of Cerebrovascular Disease, The Second Affiliated Hospital of Guangzhou University of Chinese Medicine Guangdong Provincial Hospital of Chinese Medicine Guangzhou China

**Keywords:** a body shape index, depressive symptoms, epilepsy, mediation analysis

## Abstract

**Background:**

Epilepsy is a common and disabling brain disorder. Whether a body shape index (ABSI) is associated with epilepsy, and whether depressive symptoms may partly account for this association, remain unclear.

**Methods:**

We analyzed 13,066 participants from the National Health and Nutrition Examination Survey 2013–2018. ABSI was calculated from waist circumference, weight, and height. Epilepsy was identified using prescription medication reason code G40 within the past 30 days. Depressive symptoms were assessed using Patient Health Questionnaire 9. Multivariate logistic regression was used to evaluate associations among ABSI, depressive symptoms, and epilepsy. Restricted cubic splines (RCSs) were applied to examine potential nonlinearity. An exploratory mediation analysis was performed to examine whether depressive symptoms may partly account for the observed association between ABSI and epilepsy.

**Results:**

In the multivariate logistic regression analyses, higher ABSI was significantly associated with increased odds of both epilepsy (odds ratio [OR], 1.39; 95% confidence interval [CI], 1.12–1.71) and depressive symptoms (OR, 1.16; 95% CI, 1.08–1.24). Additionally, a positive association was also identified between depressive symptoms and epilepsy (OR, 2.28; 95% CI, 1.41–3.69). RCS analysis revealed that the associations between ABSI and both epilepsy and depressive symptoms were linear (both *p* for nonlinearity > 0.05). The exploratory mediation analysis indicated that depressive symptoms may partly account for the association between ABSI and epilepsy, accounting for 10.8% of the overall association.

**Conclusions:**

Higher ABSI was associated with a higher prevalence of epilepsy, and depressive symptoms may partly account for this relationship. These findings highlight the potential importance of integrating body composition and mental health perspectives in epilepsy epidemiology and may inform future prospective studies on modifiable metabolic and psychological factors.

## Graphical abstract

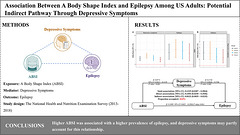

**Abbreviations**
ABSI: a body shape index
BMI: body mass index
CI: confidence interval
GBD: Global Burden Of Disease
ICD: International Classification of Diseases
NHANES: National Health and Nutrition Examination Survey
OR: odds ratio
PHQ‐9: Patient Health Questionnaire‐9
PLI: poverty level index
RCS: restricted cubic spline
SD: standard deviation
WHO: World Health Organization

## 1 Introduction

Epilepsy is a serious disorder of the brain characterized by recurrent seizures, and its disease burden remains substantial (Fisher et al. 2014; Thijs et al. 2019). According to the 2021 Global Burden of Disease (GBD) Study, the worldwide prevalence of epilepsy has reached approximately 51.7 million individuals (GBD Epilepsy Collaborators 2025). Furthermore, the World Health Organization (WHO) reports that approximately 5 million people are diagnosed with epilepsy each year (World Health Organization 2024). In addition to an elevated risk of premature mortality, epilepsy is closely associated with cognitive and affective problems, accidental injuries, and stigma and discrimination, which markedly reduce quality of life and impose sustained socioeconomic burden (Thijs et al. 2019; World Health Organization 2024; World Health Organization et al. 2019). Although current antiseizure medications can render a substantial proportion of patients seizure free, pronounced treatment gaps persist in many regions (Meyer et al. 2010; Thijs et al. 2019; World Health Organization 2024). Notably, the WHO suggests that about 25% of epilepsy cases are potentially preventable, which highlights the importance of identifying and intervening on modifiable risk factors (World Health Organization 2024).

Obesity is a well‐established risk factor for multiple chronic diseases (World Health Organization 2025), yet epidemiologic evidence linking obesity to epilepsy remains inconsistent (Choi et al. 2017; Gao et al. 2008). Some studies report a higher epilepsy risk with increasing obesity severity, whereas others suggest a nonlinear pattern with elevated risk mainly in underweight and extreme obesity (Gao et al. 2008; Razaz et al. 2017). One likely reason is that body mass index (BMI) cannot distinguish fat mass from lean mass and it provides limited information on abdominal fat distribution, which can lead to exposure misclassification and biased association estimates (Am and Sa 2001; Centers for Disease Control and Prevention 2011). As a complementary indicator of body shape and central adiposity, Krakauer proposed a body shape index (ABSI) in 2012, an allometrically scaled waist circumference metric adjusted for height and BMI (Krakauer and Krakauer 2012). Prior studies have linked a higher ABSI to adverse health outcomes, including elevated risks of all‐cause mortality, cardiovascular mortality, cancer mortality, and incident type 2 diabetes (Boonpor et al. 2023; Christakoudi et al. 2020; Dhana et al. 2016). However, the evidence relating the ABSI to epilepsy remains limited.

Depressive symptoms are among the most common and clinically consequential psychiatric comorbidities in epilepsy (Fiest et al. 2013). Meta‐analytic evidence indicates that approximately one quarter of people with epilepsy experience active depressive symptoms (Fiest et al. 2013). Large‐scale cohort and registry based studies further suggest a two‐way association between depressive symptoms and epilepsy, in which depressive symptoms may precede epilepsy onset and is associated with higher subsequent epilepsy risk, while epilepsy may also increase the subsequent risk of depressive symptoms (Bølling‐Ladegaard et al. 2023; Josephson et al. 2017). Meanwhile, recent evidence has linked higher ABSI values to a greater depressive symptoms burden (Zhang et al. 2024). Taken together, these findings support the hypothesis that depressive symptoms may play a key role in the pathway linking ABSI to epilepsy risk, while this potential mechanism requires further exploration.

To address these gaps, we leveraged a large cross‐sectional survey to examine the association between the ABSI and the prevalence of epilepsy and to further explore whether depressive symptoms may partly account for this association. These findings may provide hypothesis‐generating evidence for future prospective studies.

## 2 Methods

### 2.1 Study Design and Participants

This study used data from the National Health and Nutrition Examination Survey (NHANES). NHANES is an ongoing nationwide program that began in 1999 and is designed to evaluate the health and nutritional status of the US population (Paulose‐Ram et al. 2021). It uses a continuous multistage probability sampling design and recruits a nationally representative sample every 2 years. NHANES protocols were approved by the Research Ethics Review Board of the National Center for Health Statistics. All participants provided written informed consent before participation. The NHANES data are publicly available and de‐identified. Therefore, no additional ethical approval or administrative permission was required for the present analyses. All analyses were conducted in accordance with NHANES analytic guidance and relevant regulations (C. Johnson et al. 2013). In this study, we extracted and analyzed data from the NHANES cycles conducted from 2013 to 2018, which were maintained by the National Center for Health Statistics at the Centers for Disease Control and Prevention.

The study initially included 29,400 participants. We excluded individuals who were younger than 20 years, which included 12,343 participants. We further excluded 1766 participants with missing information on epilepsy status or ABSI. In addition, 1096 participants with missing data on depressive symptoms were excluded. Finally, 1129 participants were excluded due to missing covariate data (Table S1). The final analytic sample comprised 13,066 participants.

### 2.2 Epilepsy

The NHANES questionnaire data did not directly assess epilepsy. Instead, information on prescription medication use was collected through face‐to‐face interviews between the interviewer and participants. We adopted the same method for defining epilepsy as used in previous related studies (Huang et al. 2024; Yang et al. 2024). Participants were classified based on whether they reported taking at least one medication specifically for “epilepsy and recurrent seizures” (International Classification of Diseases [ICD] code G40). Specifically, epilepsy was identified using the ICD‐10‐CM codes for the reported reason for prescription medication use in RXDRSC1, RXDRSC2, or RXDRSC3. All participants provided the names of medications prescribed by healthcare providers in the past 30 days, along with the primary reason for using each medication.

### 2.3 Depressive Symptoms

Depressive symptoms were assessed using the 9‐item Patient Health Questionnaire (PHQ‐9), a widely used, validated self‐administered instrument for screening and grading the severity of depressive symptoms (Kroenke et al. 2001). The PHQ‐9 comprises nine symptom items (anhedonia, depressed mood, sleep disturbance, fatigue, appetite changes, feelings of worthlessness, difficulty concentrating, psychomotor agitation/retardation, and suicidal ideation). Each item is scored from 0 (“not at all”) to 3 (“nearly every day”), yielding a total score ranging from 0 to 27, with higher scores indicating more severe depressive symptoms. A total score ≥ 10 was used to indicate moderate‐to‐severe depressive symptoms, a cut‐point that demonstrated 88% sensitivity and 88% specificity in the original validation study (Kroenke et al. 2001).

### 2.4 ABSI

ABSI is a novel anthropometric indicator intended to capture body shape and central adiposity beyond overall body size by integrating waist circumference with height and weight through allometric scaling (Krakauer and Krakauer 2012). In contrast to BMI, which does not differentiate fat mass from lean mass and provides limited information on abdominal fat distribution, ABSI more directly reflects abdominal body shape (Krakauer and Krakauer 2012). In this study, ABSI was modeled as a continuous variable to evaluate its potential associations with epilepsy and depressive symptoms. ABSI was calculated as $\frac{\mathrm{Waist}\;\mathrm{Circumference}}{\mathrm{BM}I^{2/3}\times \mathrm{Heigh}t^{1/2}}$, where waist circumference and height are measured in centimeters. Given the narrow numerical distribution of ABSI, ABSI was standardized before subsequent statistical analyses.

### 2.5 Covariates

Consistent with prior literature (Razaz et al. 2017; Zhu et al. 2026), we included a set of potential confounders to reduce residual confounding. Demographic variables included age, sex (male or female), and ethnicity (Mexican American, Non‐Hispanic White, Non‐Hispanic Black, and Other). Socioeconomic factors included education level (under high school, high school or equivalent, above high school), marital status (married/living with partner, never married, widowed/divorced/separated), and income (poverty level index [PLI] ≤ 1.3, 1.3< PLI ≤ 1.85, PLI > 1.85). Behavioral and lifestyle variables included smoking status (current, never, previous), alcohol consumption (current, never, previous), and physical activity.

### 2.6 Statistical Analysis

For descriptive analyses, continuous variables are presented as mean ± standard deviation (SD), and categorical variables are presented as counts and percentages. Between group differences in continuous variables were assessed using the *t*‐test. Differences in categorical variables were assessed using the $\chi^{2}$ test.

We used multivariate logistic regression to examine pairwise associations among ABSI, depressive symptoms, and epilepsy. A sequential modeling strategy was applied. Model 1 was unadjusted. Model 2 adjusted for age, sex, and ethnicity. Model 3 further adjusted for marital status, education level, and income. Model 4 additionally adjusted for smoking status, alcohol consumption, and physical activity. Odds ratios (ORs) with 95% confidence intervals (CI) were reported for all models. Restricted cubic spline (RCS) models with three knots located at the 10th, 50th, and 90th percentiles (0.757, 0.819, and 0.881, respectively) were used to evaluate potential nonlinearity and overall trends in the associations of ABSI with depressive symptoms and epilepsy. These models were adjusted for the same covariates included in the fully adjusted logistic regression model. Using a structural equation modeling framework implemented with the R package “lavaan,” we conducted an exploratory mediation analysis to assess a potential indirect pathway of depressive symptoms in the association between ABSI and epilepsy. The mediation model was adjusted for the same covariates as the logistic regression analyses. We performed subgroup analyses to examine whether the association between ABSI and epilepsy varied across age, sex, ethnicity, income, alcohol consumption, and smoking status.

To assess the robustness of the findings, several sensitivity analyses were conducted. First, we used multiple imputation to address missing covariate data and reevaluated the pairwise associations among ABSI, depressive symptoms, and epilepsy. Second, based on the fully adjusted model, we additionally adjusted for comorbid conditions, including hypertension, diabetes, and hyperlipidemia. Third, we performed an additional analysis after excluding participants who reported using valproate or carbamazepine. Fourth, we performed a survey‐weighted sensitivity analysis accounting for the NHANES MEC examination weight, strata, and primary sampling units. Finally, we calculated *E*‐values to evaluate the potential impact of unmeasured confounding on the observed associations.

## 3 Results

### 3.1 Baseline Characteristics

In this study, 13,066 participants were included, including 106 with epilepsy. Compared with participants without epilepsy, those with epilepsy were older (53.62 vs. 49.52 years) and had higher ABSI values (0.84 vs. 0.82) and PHQ‐9 scores (5.63 vs. 3.20). Marital status and income differed markedly, with fewer married/partnered individuals (40.6% vs. 60.1%) and a higher proportion with PLI ≤ 1.3 (58.5% vs. 33.9%) in the epilepsy group. Alcohol and smoking status also differed modestly, whereas sex, ethnicity, education, and physical activity were comparable between groups. Detailed information was presented in Table 1.

**TABLE 1:** Baseline characteristics of participants according to epilepsy status.

Characteristic	Overall *N* = 13,066^a^	Non‐Epilepsy *N* = 12,960^a^	Epilepsy *N* = 106^a^	*p* ^b^
Age	49.55 ± 17.42	49.52 ± 17.43	53.62 ± 15.68	0.008
Sex				> 0.999
Female	6694 (51.2)	6640 (51.2)	54 (50.9)	
Male	6372 (48.8)	6320 (48.8)	52 (49.1)	
Ethnicity				0.222
Mexican American	1871 (14.3)	1858 (14.3)	13 (12.3)	
Non‐Hispanic White	5112 (39.1)	5061 (39.1)	51 (48.1)	
Non‐Hispanic Black	2744 (21.0)	2722 (21.0)	22 (20.8)	
Other	3339 (25.6)	3319 (25.6)	20 (18.9)	
Education				0.189
Under high school	2571 (19.7)	2548 (19.7)	23 (21.7)	
High school or equivalent	3002 (23.0)	2971 (22.9)	31 (29.2)	
Above high school	7493 (57.3)	7441 (57.4)	52 (49.1)	
Marriage				< 0.001
Married/living with partner	7829 (59.9)	7786 (60.1)	43 (40.6)	
Never married	2415 (18.5)	2382 (18.4)	33 (31.1)	
Widowed/divorced/separated	2822 (21.6)	2792 (21.5)	30 (28.3)	
Income				< 0.001
PLI ≤ 1.3	4450 (34.1)	4388 (33.9)	62 (58.5)	
1.3 < PLI ≤ 1.85	1993 (15.3)	1978 (15.3)	15 (14.2)	
PLI > 1.85	6623 (50.7)	6594 (50.9)	29 (27.4)	
Alcohol status				0.018
Current	9258 (70.9)	9193 (70.9)	65 (61.3)	
Never	1765 (13.5)	1751 (13.5)	14 (13.2)	
Previous	2043 (15.6)	2016 (15.6)	27 (25.5)	
Smoke status				0.041
Current	2540 (19.4)	2511 (19.4)	29 (27.4)	
Never	7397 (56.6)	7349 (56.7)	48 (45.3)	
Quit	3129 (23.9)	3100 (23.9)	29 (27.4)	
Physical activity	64.38 ± 107.68	64.51 ± 107.79	47.88 ± 92.79	0.069
ABSI	0.82 ± 0.05	0.82 ± 0.05	0.84 ± 0.05	< 0.001
Depressive symptoms	3.22 ± 4.24	3.20 ± 4.22	5.63 ± 5.77	< 0.001

^a^ Values are presented as mean ± standard deviation for continuous variables and as number with percentage for categorical variables.

^b^ *p* values were calculated by comparing participants with epilepsy and those without epilepsy. Continuous variables were compared using the independent samples *t* test. Categorical variables were compared using the chi square test.

Abbreviations: ABSI, a body shape index; PLI, poverty level index.

### 3.2 Associations Among ABSI, Depressive Symptoms, and Epilepsy

**FIGURE 1:**
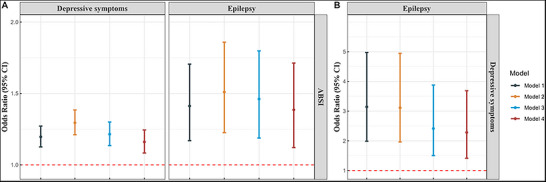
Associations among ABSI, depressive symptoms, and epilepsy across sequentially adjusted models. Panel A shows the associations of ABSI with depressive symptoms and with epilepsy. Panel B shows the association of depressive symptoms with epilepsy. Points represent odds ratios and vertical lines represent 95% confidence intervals from logistic regression models. The horizontal reference line indicates an odds ratio of 1. Model 1 was unadjusted. Model 2 adjusted for age, sex, and ethnicity. Model 3 further adjusted for marital status, education level, and household income. Model 4 additionally adjusted for smoking status, alcohol consumption, and physical activity. Odds ratios for ABSI are presented per 1 standard deviation increase. ABSI, a body shape index.

Figure 1 showed that higher ABSI was positively associated with both epilepsy and depressive symptoms across all models. For example, in the fully adjusted model (Model 4), each 1 SD increase in ABSI was linked to higher odds of epilepsy (OR = 1.39, 95% CI: 1.12–1.71; *p* = 0.003) and depressive symptoms (OR = 1.16, 95% CI: 1.08–1.24; *p* < 0.001). Detailed information was presented in Table S2.

### 3.3 Nonlinear Relationship Between ABSI and Depressive Symptoms, and Epilepsy

**FIGURE 2:**
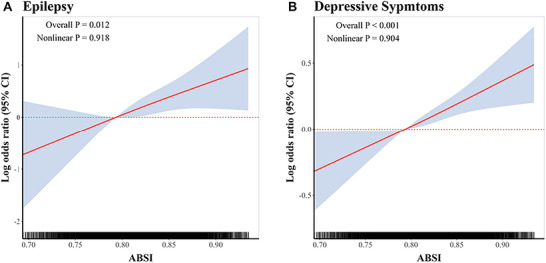
Restricted cubic spline analyses of the associations of ABSI with epilepsy and depressive symptoms. Restricted cubic spline models with three knots located at the 10th, 50th, and 90th percentiles (0.757, 0.819, and 0.881, respectively) were used to evaluate potential nonlinearity in the associations between ABSI and epilepsy or depressive symptoms. Panel A shows epilepsy as the outcome and Panel B shows depressive symptoms as the outcome. The solid line represents the adjusted estimate of the log odds ratio and the shaded area represents the 95% confidence interval. Vertical dotted lines indicate knot locations, and the rug plot along the *x* axis shows the distribution of ABSI. Models were adjusted for the same covariates as the fully adjusted logistic regression model. ABSI, a body shape index.

Figure 2 showed positive and approximately linear associations of ABSI with both epilepsy and depressive symptoms in the fully adjusted model. For epilepsy, the overall association was significant (overall *p* = 0.012), with no evidence of nonlinearity (*p* for nonlinearity = 0.918). Similarly, ABSI was significantly associated with depressive symptoms (overall *p* < 0.001), and the nonlinearity test was not significant (*p* for nonlinearity = 0.904).

### 3.4 Mediation Analysis

**FIGURE 3:**
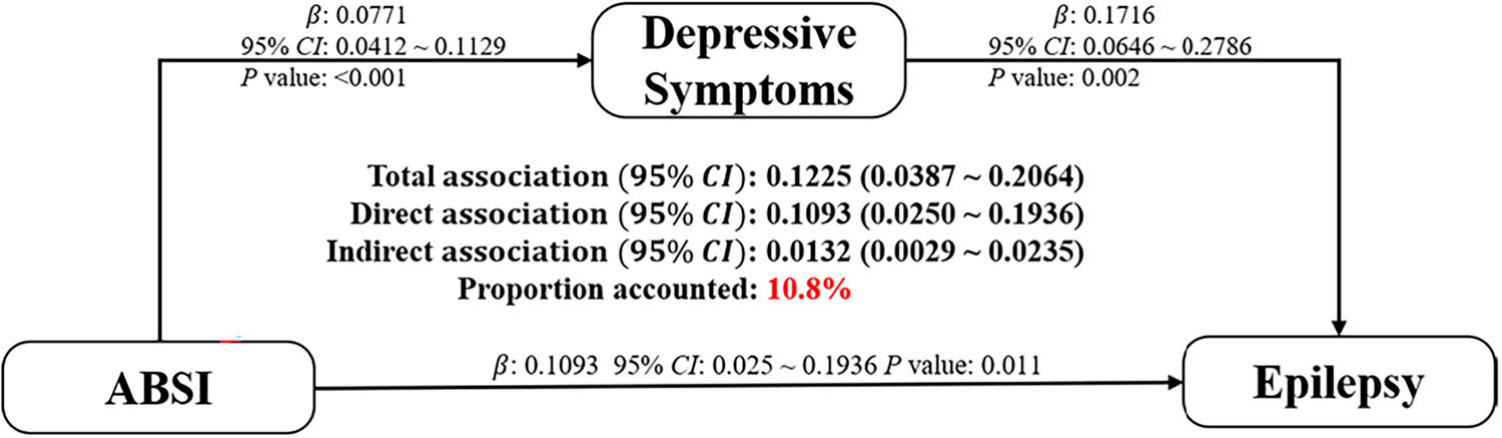
Exploratory analysis of the potential indirect pathway through depressive symptoms in the association between ABSI and epilepsy. The model included ABSI as the exposure, depressive symptoms as the potential intermediate variable, and epilepsy as the outcome. The reported estimates represent the total, direct, and indirect associations. The potential indirect pathway through depressive symptoms accounted for 10.8% of the overall association. The model was estimated using structural equation modeling and adjusted for the same covariates as the fully adjusted logistic regression model. ABSI, a body shape index.

Figure 3 presented that depressive symptoms may partly account for the association between ABSI and epilepsy. The total association was 0.1225 (95% CI: 0.0387–0.2064), the direct association was 0.1093 (95% CI: 0.0250–0.1936), and the indirect association through depressive symptoms was 0.0132 (95% CI: 0.0029–0.0235). The indirect pathway through depressive symptoms accounted for 10.8% of the overall association between ABSI and epilepsy.

### 3.5 Subgroup Analysis

**FIGURE 4:**
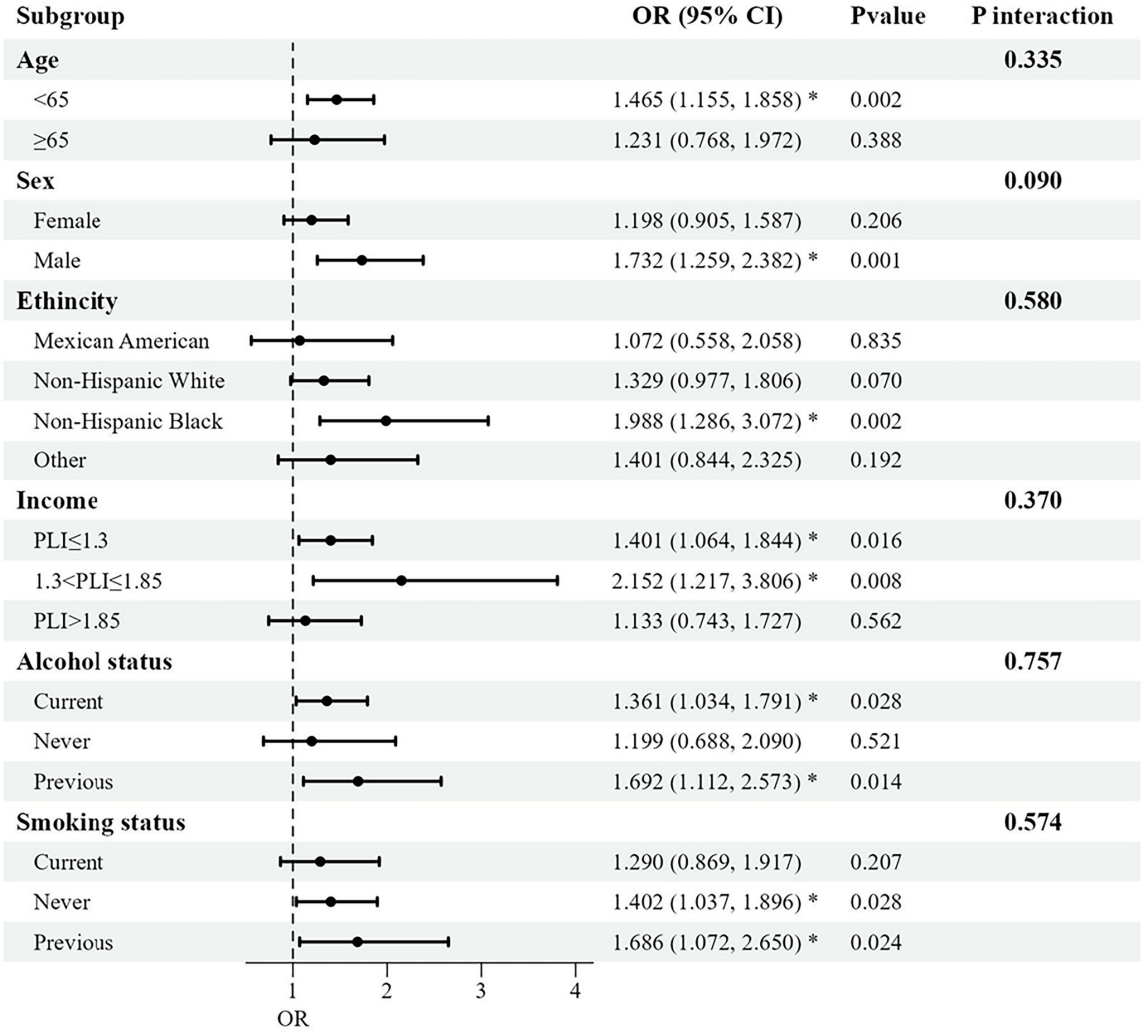
Subgroup analyses of the association between ABSI and epilepsy. Forest plot of subgroup analyses for the association between ABSI and epilepsy. Odds ratios (OR) and 95% confidence intervals (CI) are shown for each subgroup, estimated from logistic regression models adjusted for the covariates in the fully adjusted model. *p* values correspond to within subgroup associations, and *p* for interaction values test multiplicative interaction terms between ABSI and the subgroup variable. ABSI, a body shape index; PLI, poverty level index.

Figure 4 showed that higher ABSI was generally associated with higher odds of epilepsy, with statistically significant associations observed among participants aged < 65 years (OR 1.47, 95% CI 1.16–1.86), males (OR 1.73, 95% CI 1.26–2.38), non‐Hispanic Black participants (OR 1.99, 95% CI 1.29–3.07), those with lower/middle income (PLI ≤ 1.3: OR 1.40, 95% CI 1.06–1.84; 1.3 < PLI ≤ 1.85: OR 2.15, 95% CI 1.22–3.81), current or previous drinkers (OR 1.36 and 1.69), and never or former smokers (OR 1.40 and 1.69). There was no evidence of effect modification by age, sex, ethnicity, income, alcohol status, or smoking status (all *p* for interaction > 0.05). The numeric results were presented in Table S3.

### 3.6 Sensitivity Analysis

Sensitivity analyses yielded generally consistent results. Using multiple imputation, the associations among ABSI, depressive symptoms, and epilepsy were essentially unchanged (Table S4). Further adjustment for hypertension, diabetes, and hyperlipidemia slightly attenuated the associations, but the results remained statistically significant (Table S5). After excluding participants who reported using valproate or carbamazepine, the results remained consistent with the main analysis (Table S6). In the survey‐weighted sensitivity analysis, the associations were generally consistent in direction with the main analysis (Table S7). The *E*‐values for the fully adjusted estimates were 2.12, 1.59, and 3.99, respectively, suggesting that unmeasured confounding would be required to explain away the observed associations (Table S8).

## 4 Discussion

Based on a large nationally representative cross‐sectional survey conducted in the United States, our research has identified a positive association between ABSI and epilepsy. Concurrently, we observed a linear association between ABSI and epilepsy. Furthermore, depressive symptoms may partly account for the association between ABSI and epilepsy. To our knowledge, this study adds additional epidemiological evidence on the association between ABSI and epilepsy, with a particular focus on the potential indirect role of depressive symptoms.

### 4.1 Comparison With Previous Studies

Our research has identified that ABSI may be associated with increased odds of epilepsy. Although direct evidence linking ABSI to epilepsy is currently lacking, ABSI is calculated using height and waist circumference, thereby indirectly representing obesity (Bertoli et al. 2017; Krakauer and Krakauer 2012). Our findings align with the majority of existing studies, which suggest that obese individuals may be more susceptible to epilepsy (Chen et al. 2021; Ladino et al. 2014; Zhou et al. 2022). For instance, a case‐control study suggested that obesity was associated with an increased prevalence of drug‐resistant epilepsy (Chen et al. 2021). A two‐sample Mendelian randomization study indicated that obesity constitutes a risk factor for epilepsy, with increased waist circumference being particularly associated with juvenile myoclonic epilepsy (Zhou et al. 2022). Furthermore, maternal obesity has been demonstrated to be linked to epilepsy in offspring (Razaz et al. 2017). However, our findings also diverge from previous studies. For instance, Gao et al.’s (2008) analysis of UK primary care databases showed that the epilepsy was similar between generally obese individuals (BMI ≥ 30 kg/m^2^) and those of normal weight, with increased seizure primarily observed in underweight (< 18.5 kg/m^2^) or severely obese (≥ 40 kg/m^2^) populations. Conversely, a large German cohort study demonstrated a significant association between low body weight and epilepsy, with a stronger effect observed in males, whereas overweight and obesity were not reported as significant associated factors (Pfeifer et al. 2022). Existing research on the obesity–epilepsy relationship yields inconsistent conclusions, potentially stemming from variations in exposure and outcome definitions, population heterogeneity (Gao et al. 2008; Nuttall 2015; Pfeifer et al. 2022), and confounding biases such as residual confounders, overadjustment, and bidirectional relationships involving reverse causality and weight gain associated with epilepsy itself or antiepileptic drugs (E. Johnson et al. 2018; Pfeifer et al. 2022; Verrotti et al. 2011; Zhou et al. 2022). Therefore, longitudinal studies employing standardized outcome definitions and causal inference methods remain necessary to clarify directionality and identify key biological pathways. In this study, we observed an association between increased ABSI and higher epilepsy prevalence. As ABSI is calculated using height and waist circumference, our findings align more closely with those of Zhou et al. (2022) and Zhu et al. (2026). However, our observational cross‐sectional design precludes establishing causality between ABSI and epilepsy. Future studies across diverse populations are required to validate these findings.

The clinical interpretation of ABSI should be cautious. ABSI was originally developed as a body‐shape indicator related to waist circumference beyond BMI and has been evaluated in relation to mortality, cardiometabolic outcomes, and visceral adiposity‐related risk (Bertoli et al. 2017; Bouchi et al. 2016; Krakauer and Krakauer 2012). However, unlike BMI, ABSI does not currently have universally accepted clinical cutoffs, particularly for epilepsy, and its interpretation is usually outcome‐ and population‐dependent (Christakoudi et al. 2020; Dhana et al. 2016). Therefore, although participants with epilepsy had a statistically higher mean ABSI than those without epilepsy (0.84 vs. 0.82), this modest absolute difference should be interpreted as a population‐level association rather than a stand‐alone clinically diagnostic difference.

### 4.2 Potential Mechanisms

Because ABSI partly reflects abdominal and potentially visceral adiposity, several pathways reported in previous studies may provide biologically plausible explanations for the observed association between ABSI and epilepsy (Bouchi et al. 2016; Krakauer and Krakauer 2012; Rhea et al. 2017). First, visceral adiposity is closely related to chronic low‐grade inflammation. Pro‐inflammatory cytokines and obesity‐related blood‐brain barrier dysfunction may promote neuroinflammatory responses, which have been implicated in altered neuronal excitability and epilepsy‐related processes (Feng et al. 2024; Vezzani et al. 2013). Second, obesity‐related metabolic disturbances and adipokine imbalance may be linked to seizure susceptibility by influencing energy metabolism and neurotransmitter regulation. For example, previous studies have suggested that leptin and adiponectin signaling may be involved in epilepsy‐related metabolic and neuronal excitability pathways (Lee et al. 2011; Shan et al. 2023; Shanley et al. 2002). Third, selected neurobiological changes may also be relevant. Overweight and obesity have been associated with smaller hippocampal volumes and faster hippocampal atrophy, while hippocampal sclerosis is an important pathological substrate of temporal lobe epilepsy (Blümcke et al. 2013; Cherbuin et al. 2015). In addition, gut microbiota alterations have been reported in epilepsy, and experimental evidence suggests that the antiseizure effects of the ketogenic diet may be partly mediated by gut microbiota (Gong et al. 2020; Olson et al. 2018). Overall, these mechanisms should be regarded as evidence‐informed hypotheses rather than pathways directly demonstrated by the present cross‐sectional study. Future longitudinal and mechanistic studies incorporating inflammatory biomarkers, adipokines, neuroimaging markers, mitochondrial function, and gut microbiota are needed to clarify these potential mechanisms.

### 4.3 Mediation Analysis

Our study also explored whether depressive symptoms may partly account for the observed association between ABSI and epilepsy. Previous studies have consistently demonstrated that individuals with elevated ABSI exhibit markedly severe depressive symptoms. Obesity and depressive symptoms share multiple immunometabolic pathways, including mechanisms involving leptin, insulin signaling, and the microbiota (Lotfi et al. 2022; Milaneschi et al. 2019; Zhang et al. 2024). There is a growing body of evidence suggesting that depressive symptoms and epilepsy share common underlying pathophysiological mechanisms, including inflammation and dysregulation of the hypothalamic‐pituitary‐adrenal axis (Hooper et al. 2018; Shi et al. 2025). Depressive symptoms have been linked to epilepsy and worsening of seizure control, as well as a predictor of poorer outcomes in individuals with epilepsy (Bølling‐Ladegaard et al. 2023; Josephson et al. 2017). It is well established that obesity, particularly abdominal fat accumulation, is strongly associated with depressive symptoms, likely due to the inflammatory and metabolic disruptions associated with excessive adiposity (Hryhorczuk et al. 2013). Our findings suggest that depressive symptoms may represent a potential indirect pathway in the association between ABSI and epilepsy. This aligns with the hypothesis that the chronic inflammatory and metabolic obesity‐related changes may be associated with both depressive symptoms and epilepsy by altering neuronal excitability and increasing the frequency of seizures (Li et al. 2024). This pathway provides a potential explanation for how ABSI‐related metabolic and inflammatory disturbances may be associated with both depressive symptoms and epilepsy. Because ABSI, depressive symptoms, and epilepsy were assessed within a cross‐sectional framework, the mediation analysis cannot establish temporal ordering or causal mediation. Therefore, depressive symptoms should be interpreted as a potential indirect pathway rather than a confirmed mediator.

### 4.4 Strengths and Limitations

Our study has several strengths. First, this analysis was conducted in a large nationally representative population‐based cross‐sectional survey from the United States, with a substantial sample size and broad population coverage, which enhances the stability and precision of effect estimates. Second, we systematically adjusted for a wide range of key confounding factors, including demographic characteristics, socioeconomic status, and lifestyle factors, and further evaluated the robustness of our findings through multiple sensitivity analyses. Third, we used the ABSI as the primary exposure, which may better capture abdominal adiposity and body fat distribution than the conventional BMI. Finally, to our knowledge, this study is among the first to report an association between ABSI and epilepsy at the population level and to further evaluate the shape of this association, providing hypothesis‐generating evidence for future research.

Several limitations should also be acknowledged. First, the observational cross‐sectional design precludes causal inference and does not allow determination of temporal ordering between ABSI, depressive symptoms, and epilepsy. Therefore, the mediation analysis should be interpreted as exploratory, because this design could not verify whether ABSI preceded depressive symptoms or whether depressive symptoms preceded epilepsy. Future studies based on larger prospective cohorts and diverse populations are needed to confirm these findings. Second, epilepsy was identified using ICD‐10‐CM medication reason codes from NHANES prescription medication data rather than standardized clinical diagnostic criteria. Although this definition was based on the reported reason for medication use rather than medication name alone, outcome misclassification may still exist because NHANES does not provide information on seizure type, neurologist‐confirmed diagnosis, EEG findings, seizure frequency, age at onset, or epilepsy duration. In addition, certain antiseizure medications may affect body weight or body fat distribution. Although we performed a sensitivity analysis excluding valproate or carbamazepine users, NHANES lacks detailed information on medication duration and dose, preventing reliable medication‐specific analyses. Third, depressive symptoms were assessed using the PHQ‐9 questionnaire without clinical diagnostic confirmation, which may introduce measurement error and affect the estimation of the potential indirect pathway. In addition, we were unable to adjust for the potential influence of psychotropic medications, such as tricyclic antidepressants and antipsychotics, which may introduce medication‐related residual confounding. Fourth, although we adjusted for multiple important confounders, residual confounding cannot be entirely excluded. Unmeasured factors such as genetic susceptibility to epilepsy, environmental exposures, and other clinical characteristics may partially influence the observed association between ABSI and epilepsy. In addition, because no formal adjustment for multiple comparisons was performed, the possibility of type I error cannot be excluded. Future longitudinal studies incorporating more comprehensive epilepsy ascertainment and detailed clinical information, including seizure type and frequency, age at onset, electroencephalographic or neuroimaging findings, and antiseizure medication profiles, are warranted to further clarify the underlying mechanisms.

## 5 Conclusions

In summary, our study offers important insights into the association among ABSI, depressive symptoms, and epilepsy. Higher ABSI was positively associated with epilepsy, and depressive symptoms may partly account for this observed association. Future longitudinal and mechanistic studies are needed to confirm these associations, clarify temporal relationships, and further investigate the potential biological and psychological pathways linking ABSI, depressive symptoms, and epilepsy.

## Author Contributions

**Xiujuan Mi**: conceptualization, methodology, data curation, formal analysis, writing – review and editing. **Jinsong You**: supervision, Writing – review and editing. **Qiaoduan Feng**: conceptualization, methodology, data curation, validation, formal analysis, visualization, writing – original draft, writing – review and editing, software. **Jun Tang**: supervision, writing – review and editing, conceptualization, methodology, funding acquisition, resources, project administration. **Hongbin Tang**: conceptualization, writing – review and editing, formal analysis, investigation. **Can Wan**: writing – review and editing, validation. **Shaokun Yang**: formal analysis, writing – review and editing.

## Funding

This study was supported by the Natural Science Foundation Project of Chongqing, Chongqing Science and Technology Commission, China (No. cstc2019jcyj‐msxmX0688) and Chongqing Bayu Qihuang Scholars Support Project (Chongqing Traditional Chinese Medicine [2023] No. 23).

## Ethics Statement

All procedures performed in studies involving human participants were in accordance with the ethical standards of the institutional and/or national research committee, the 1964 Helsinki Declaration and its later amendments, or comparable ethical standards. The study received ethical clearance from the Institutional Review Board of National Center for Health Statistics. Informed consent was obtained from all individual participants prior to data collection.

## Conflicts of Interest

The authors declare no conflicts of interest.

## Supporting information




**Supplementary Table S1‐S8**: brb371625‐sup‐0001‐TableS1‐S8.docx

## Data Availability

Publicly available datasets were analyzed in this study. This data can be found at https://www.cdc.gov/nchs/nhanes/index.htm.
