# Supplementary material for: Association Between a Body Shape Index and Epilepsy Among US Adults: Potential Indirect Pathway Through Depressive Symptoms
Source: Brain Behav. 2026 Jul 28;16(8):e71625. doi: 10.1002/brb3.71625 (PMC13410275; doi:10.1002/brb3.71625)
Supplement: Supplementary file 1 — Supplementary Table S1‐S8: brb371625‐sup‐0001‐TableS1‐S8.docx [file BRB3-16-e71625-s001.docx]

**Supplementary Materials**

**A Body Shape Index and Epilepsy in US Adults: The Mediating Role of Depressive Symptoms**

# Contents

[Contents 2](#_Toc232373083)

[Supplementary Materials 3](#_Toc232373084)

[Table S1 Associations among a body shape index, depressive symptoms, and epilepsy 3](#_Toc232373085)

[Table S2 Subgroup analyses of the association between a body shape index and epilepsy 4](#_Toc232373086)

[Table S3 Sensitivity analysis for the associations among a body shape index, depressive symptoms, and epilepsy after multiple imputation for missing covariates 5](#_Toc232373087)

[Table S4 Table S4 Sensitivity analysis for the associations among a body shape index, depressive symptoms, and epilepsy after additionally adjusting for medical conditions 6](#_Toc232373088)

[Table S5 Sensitivity analysis for the associations among a body shape index, depressive symptoms, and epilepsy after excluding participants who reported using valproate or carbamazepine 7](#_Toc232373089)

[Table S6 Survey-weighted sensitivity analysis for the associations among A Body Shape Index, depressive symptoms, and epilepsy 8](#_Toc232373090)

[Table S7 Evalues for the associations among a body shape index, depressive symptoms, and epilepsy 9](#_Toc232373091)

# Supplementary Materials

**Table S1 Missingness of covariates among participants with complete data on epilepsy, depressive symptoms, and ABSI (N = 14,195)**

| Variable | N | Missing, n | Missing, % |
| --- | --- | --- | --- |
| Epilepsy | 14195 | 0 | 0.00 |
| Depressive symptoms | 14195 | 0 | 0.00 |
| ABSI | 14195 | 0 | 0.00 |
| Age | 14195 | 0 | 0.00 |
| Sex | 14195 | 0 | 0.00 |
| Ethnicity | 14195 | 0 | 0.00 |
| Education | 14195 | 8 | 0.06 |
| Marital status | 14195 | 6 | 0.04 |
| Income | 14195 | 1,029 | 7.25 |
| Alcohol status | 14195 | 23 | 0.16 |
| Smoking status | 14195 | 7 | 0.05 |
| Physical activity | 14195 | 87 | 0.61 |

**Table S2 Associations among a body shape index, depressive symptoms, and epilepsy**

| **Outcome** | **Exposure** | **Model** | **OR (95%CI)** | ***P*** |
| --- | --- | --- | --- | --- |
| Epilepsy | ABSI | Model 1 | 1.412(1.169,1.705) | <0.001 |
| Epilepsy | ABSI | Model 2 | 1.509(1.225,1.859) | <0.001 |
| Epilepsy | ABSI | Model 3 | 1.461(1.188,1.798) | <0.001 |
| Epilepsy | ABSI | Model 4 | 1.385(1.121,1.713) | 0.003 |
| Depressive symptoms | ABSI | Model 1 | 1.196(1.125,1.271) | <0.001 |
| Depressive symptoms | ABSI | Model 2 | 1.295(1.211,1.384) | <0.001 |
| Depressive symptoms | ABSI | Model 3 | 1.214(1.135,1.300) | <0.001 |
| Depressive symptoms | ABSI | Model 4 | 1.161(1.083,1.244) | <0.001 |
| Epilepsy | Depressive symptoms | Model 1 | 3.143(1.986,4.974) | <0.001 |
| Epilepsy | Depressive symptoms | Model 2 | 3.114(1.961,4.947) | <0.001 |
| Epilepsy | Depressive symptoms | Model 3 | 2.413(1.502,3.879) | <0.001 |
| Epilepsy | Depressive symptoms | Model 4 | 2.281(1.412,3.686) | 0.001 |

Model 1 was unadjusted. Model 2 adjusted for age, sex, and ethnicity. Model 3 further adjusted for marital status, education level, and household income. Model 4 additionally adjusted for smoking status, alcohol consumption, and physical activity. Abbreviation: ABSI, a body shape index; OR, odds ratio; CI, confidence interval.

**Table S3 Subgroup analyses of the association between a body shape index and epilepsy**

| **Subgroup** | **OR (95%CI)** | ***P*** | ***P*_int_** |
| --- | --- | --- | --- |
| **Age** |  |  |  |
| <65 | 1.465(1.155,1.858) | 0.002 | 0.335 |
| ≥65 | 1.231(0.768,1.972) | 0.388 |  |
| **Gender** |  |  |  |
| Female | 1.198(0.905,1.587) | 0.206 | 0.090 |
| Male | 1.732(1.259,2.382) | 0.001 |  |
| **Ethnicity** |  |  |  |
| Mexican American | 1.072(0.558,2.058) | 0.835 | 0.580 |
| Non-Hispanic White | 1.329(0.977,1.806) | 0.07 |  |
| Non-Hispanic Black | 1.988(1.286,3.072) | 0.002 |  |
| Other | 1.401(0.844,2.325) | 0.192 |  |
| **Income** |  |  |  |
| PLI ≤ 1.3 | 1.401(1.064,1.844) | 0.016 | 0.370 |
| 1.3<PLI ≤ 1.85 | 2.152(1.217,3.806) | 0.008 |  |
| PLI>1.85 | 1.133(0.743,1.727) | 0.562 |  |
| **Alcohol status** |  |  |  |
| Current | 1.361(1.034,1.791) | 0.028 | 0.757 |
| Never | 1.199(0.688,2.090) | 0.521 |  |
| Previous | 1.692(1.112,2.573) | 0.014 |  |
| **Smoking status** |  |  |  |
| Current | 1.290(0.869,1.917) | 0.207 | 0.574 |
| Never | 1.402(1.037,1.896) | 0.028 |  |
| Quit | 1.686(1.072,2.650) | 0.024 |  |

Model adjusted for age, sex, ethnicity, marital status, education level, household income, smoking status, alcohol consumption, and physical activity. Abbreviation: ABSI, a body shape index; OR, odds ratio; CI, confidence interval.

**Table S4 Sensitivity analysis for the associations among a body shape index, depressive symptoms, and epilepsy after multiple imputation for missing covariates**

| **Outcome** | **Exposure** | **Model** | **OR (95% CI)** | ***P*** |
| --- | --- | --- | --- | --- |
| Epilepsy | ABSI | Model 1 | 1.413(1.171,1.707) | <0.001 |
| Epilepsy | ABSI | Model 2 | 1.510(1.226,1.860) | <0.001 |
| Epilepsy | ABSI | Model 3 | 1.462(1.189,1.799) | <0.001 |
| Epilepsy | ABSI | Model 4 | 1.385(1.120,1.712) | 0.003 |
| Depressive symptoms | ABSI | Model 1 | 1.193(1.122,1.268) | <0.001 |
| Depressive symptoms | ABSI | Model 2 | 1.291(1.207,1.380) | <0.001 |
| Depressive symptoms | ABSI | Model 3 | 1.211(1.131,1.296) | <0.001 |
| Depressive symptoms | ABSI | Model 4 | 1.161(1.083,1.245) | <0.001 |
| Epilepsy | Depressive symptoms | Model 1 | 3.156(1.994,4.994) | <0.001 |
| Epilepsy | Depressive symptoms | Model 2 | 3.126(1.968,4.966) | <0.001 |
| Epilepsy | Depressive symptoms | Model 3 | 2.424(1.508,3.896) | <0.001 |
| Epilepsy | Depressive symptoms | Model 4 | 2.277(1.407,3.685) | 0.001 |

Model 1 was unadjusted. Model 2 adjusted for age, sex, and ethnicity or ethnicity. Model 3 further adjusted for marital status, education level, and household income. Model 4 additionally adjusted for smoking status, alcohol consumption, and physical activity. Abbreviation: ABSI, a body shape index; OR, odds ratio; CI, confidence interval.

**Table S5 Sensitivity analysis for the associations among a body shape index, depressive symptoms, and epilepsy after additionally adjusting for medical conditions**

| **Outcome** | **Exposure** | **Model** | **OR (95% CI)** | ***P*** |
| --- | --- | --- | --- | --- |
| Epilepsy | ABSI | Model 1 | 1.413(1.171,1.707) | <0.001 |
| Epilepsy | ABSI | Model 2 | 1.510(1.226,1.860) | <0.001 |
| Epilepsy | ABSI | Model 3 | 1.462(1.189,1.799) | <0.001 |
| Epilepsy | ABSI | Model 4 | 1.385(1.120,1.712) | 0.003 |
| Epilepsy | ABSI | Model 5 | 1.268(1.020,1.577) | 0.033 |
| Depressive symptoms | ABSI | Model 1 | 1.193(1.122,1.268) | <0.001 |
| Depressive symptoms | ABSI | Model 2 | 1.291(1.207,1.380) | <0.001 |
| Depressive symptoms | ABSI | Model 3 | 1.211(1.131,1.296) | <0.001 |
| Depressive symptoms | ABSI | Model 4 | 1.161(1.083,1.245) | <0.001 |
| Depressive symptoms | ABSI | Model 5 | 1.101(1.025,1.182) | 0.008 |
| Epilepsy | Depressive symptoms | Model 1 | 3.156(1.994,4.994) | <0.001 |
| Epilepsy | Depressive symptoms | Model 2 | 3.126(1.968,4.966) | <0.001 |
| Epilepsy | Depressive symptoms | Model 3 | 2.424(1.508,3.896) | <0.001 |
| Epilepsy | Depressive symptoms | Model 4 | 2.277(1.407,3.685) | 0.001 |
| Epilepsy | Depressive symptoms | Model 5 | 1.962(1.204,3.197) | 0.007 |

Model 1 was unadjusted. Model 2 adjusted for age, sex, and ethnicity. Model 3 further adjusted for marital status, education level, and household income. Model 4 additionally adjusted for smoking status, alcohol consumption, and physical activity. Model 4 additionally adjusted for hypertension, diabetes, and hyperlipidemia. Abbreviation: ABSI, a body shape index; OR, odds ratio; CI, confidence interval.

**Table S6 Sensitivity analysis for the associations among a body shape index, depressive symptoms, and epilepsy after excluding participants who reported using valproate or carbamazepine**

| **Outcome** | **Exposure** | **Model** | **OR (95% CI)** | ***P*** |
| --- | --- | --- | --- | --- |
| Epilepsy | ABSI | Model 1 | 1.460(1.202,1.774) | <0.001 |
| Epilepsy | ABSI | Model 2 | 1.558(1.257,1.932) | <0.001 |
| Epilepsy | ABSI | Model 3 | 1.500(1.211,1.857) | <0.001 |
| Epilepsy | ABSI | Model 4 | 1.422(1.142,1.770) | 0.002 |
| Depressive symptoms | ABSI | Model 1 | 1.198(1.126,1.273) | <0.001 |
| Depressive symptoms | ABSI | Model 2 | 1.296(1.212,1.386) | <0.001 |
| Depressive symptoms | ABSI | Model 3 | 1.216(1.136,1.302) | <0.001 |
| Depressive symptoms | ABSI | Model 4 | 1.165(1.087,1.250) | <0.001 |
| Epilepsy | Depressive symptoms | Model 1 | 3.448(2.168,5.482) | <0.001 |
| Epilepsy | Depressive symptoms | Model 2 | 3.410(2.136,5.442) | <0.001 |
| Epilepsy | Depressive symptoms | Model 3 | 2.634(1.630,4.255) | <0.001 |
| Epilepsy | Depressive symptoms | Model 4 | 2.472(1.520,4.021) | 0.001 |

Model 1 was unadjusted. Model 2 adjusted for age, sex, and ethnicity. Model 3 further adjusted for marital status, education level, and household income. Model 4 additionally adjusted for smoking status, alcohol consumption, and physical activity. Abbreviation: ABSI, a body shape index; OR, odds ratio; CI, confidence interval.

**Table S7 Survey-weighted sensitivity analysis for the associations among A Body Shape Index, depressive symptoms, and epilepsy**

| **Outcome** | **Exposure** | **Model** | **OR (95% CI)** | ***P*** |
| --- | --- | --- | --- | --- |
| Epilepsy | ABSI | Model 1 | 1.234 (1.054-1.444) | 0.012 |
| Epilepsy | ABSI | Model 2 | 1.345 (1.120-1.616) | 0.003 |
| Epilepsy | ABSI | Model 3 | 1.284 (1.084-1.522) | 0.006 |
| Epilepsy | ABSI | Model 4 | 1.179 (0.986-1.410) | 0.078 |
| Depressive symptoms | ABSI | Model 1 | 1.159 (1.055-1.274) | 0.004 |
| Depressive symptoms | ABSI | Model 2 | 1.253 (1.139-1.379) | ,<0.001 |
| Depressive symptoms | ABSI | Model 3 | 1.172 (1.065-1.291) | 0.002 |
| Depressive symptoms | ABSI | Model 4 | 1.082 (0.978-1.198) | 0.134 |
| Epilepsy | Depressive symptoms | Model 1 | 2.837 (1.478-5.447) | 0.003 |
| Epilepsy | Depressive symptoms | Model 2 | 2.905 (1.521-5.546) | 0.002 |
| Epilepsy | Depressive symptoms | Model 3 | 2.034 (1.022-4.045) | 0.049 |
| Epilepsy | Depressive symptoms | Model 4 | 1.953 (0.977-3.906) | 0.065 |

Model 1 was unadjusted. Model 2 adjusted for age, sex, and ethnicity. Model 3 further adjusted for marital status, education level, and household income. Model 4 additionally adjusted for smoking status, alcohol consumption, and physical activity. Abbreviation: ABSI, a body shape index; OR, odds ratio; CI, confidence interval.

**Table S8 Evalues for the associations among a body shape index, depressive symptoms, and epilepsy**

| **Outcome** | **Exposure** | **Model** | **OR (95% CI)** | ***P*** | **Evalue** |
| --- | --- | --- | --- | --- | --- |
| Epilepsy | ABSI | Model 1 | 1.412(1.169,1.705) | <0.001 | 2.18 |
| Epilepsy | ABSI | Model 2 | 1.509(1.225,1.859) | <0.001 | 2.39 |
| Epilepsy | ABSI | Model 3 | 1.461(1.188,1.798) | <0.001 | 2.28 |
| Epilepsy | ABSI | Model 4 | 1.385(1.121,1.713) | 0.003 | 2.12 |
| Depressive symptoms | ABSI | Model 1 | 1.196(1.125,1.271) | <0.001 | 1.68 |
| Depressive symptoms | ABSI | Model 2 | 1.295(1.211,1.384) | <0.001 | 1.91 |
| Depressive symptoms | ABSI | Model 3 | 1.214(1.135,1.300) | <0.001 | 1.72 |
| Depressive symptoms | ABSI | Model 4 | 1.161(1.083,1.244) | <0.001 | 1.59 |
| Epilepsy | Depressive symptoms | Model 1 | 3.143(1.986,4.974) | <0.001 | 5.74 |
| Epilepsy | Depressive symptoms | Model 2 | 3.114(1.961,4.947) | <0.001 | 5.68 |
| Epilepsy | Depressive symptoms | Model 3 | 2.413(1.502,3.879) | <0.001 | 4.26 |
| Epilepsy | Depressive symptoms | Model 4 | 2.281(1.412,3.686) | 0.001 | 3.99 |

Model adjusted for age, sex, ethnicity, marital status, education level, household income, smoking status, alcohol consumption, and physical activity. Abbreviation: ABSI, a body shape index; OR, odds ratio; CI, confidence interval.
